# Simultaneous Dual-Mode Emission and Tunable Multicolor in the Time Domain from Lanthanide-Doped Core-Shell Microcrystals

**DOI:** 10.3390/nano8121023

**Published:** 2018-12-07

**Authors:** Dandan Ju, Feng Song, Adnan Khan, Feifei Song, Aihua Zhou, Xiaoli Gao, Huimin Hu, Xu Sang, Victor Zadkov

**Affiliations:** 1 School of Physics & The Key Laboratory of Weak Light Nonlinear Photonics, Ministry of Education, Nankai University, Tianjin 300071, China; judandan@mail.nankai.edu.cn (D.J.); adnanphyzx@mail.nankai.edu.cn (A.K.); songff@mail.nankai.edu.cn (F.S.); zhouihua@mail.nankai.edu.cn (A.Z.); 1120180066@mail.nankai.edu.cn (X.G.); 2120170177@mail.nankai.edu.cn (H.H.); 2120180202@mail.nankai.edu.cn (X.S.); 2 Collaborative Innovation Center of Extreme Optics, Shanxi University, Taiyuan 030006, Shanxi, China; 3 The Institute of Spectroscopy of the Russian Academy of Sciences, Moscow 142190, Russia; zadkov@isan.troitsk.ru

**Keywords:** NaYF_4_:Yb/Er@NaYF_4_:Ce/Tb/Eu microrods, dual-mode emission, time-resolved emission spectra, multicolor emission

## Abstract

The dual-mode emission and multicolor outputs in the time domain from core-shell microcrystals are presented. The core-shell microcrystals, with NaYF_4_:Yb/Er as the core and NaYF_4_:Ce/Tb/Eu as the shell, were successfully fabricated by employing the hydrothermal method, which confines the activator ions into a separate region and minimizes the effect of surface quenching. The material is capable of both upconversion and downshifting emission, and their multicolor outputs in response to 980 nm near-infrared (NIR) excitation laser and 252 nm, and 395 nm ultraviolet (UV) excitation light have been investigated. Furthermore, the tunable color emissions by controlling the Tb^3+^-Eu^3+^ ratio in shells and the energy transfer of Ce^3+^→Tb^3+^→Eu^3+^ were discussed in details. In addition, color tuning of core-shell-structured microrods from green to red region in the time domain could be obtained by setting suitable delay time. Due to downshifting multicolor outputs (time-resolved and pump-wavelength-induced downshifting) coupled with the upconversion mode, the core-shell microrods can be potentially applied to displays and high-level security.

## 1. Introduction

Lanthanide (Ln^3+^)-doped luminescent materials with various compositions and properties have been well studied for the last few decades. Compared with traditional luminescent materials, such as quantum dots (QDs) and organic dyes, the lanthanide-doped nano- and micro-crystals show more superior optical features including narrow bands widths, stable energy levels, low toxicity and long lifetime [1,2]. In addition, these materials have attracted a great deal of attention owing to their magnetic and thermal properties. These properties lead to widespread applications in various areas, such as volumetric display [3,4], solar cell [5], optical data storage [3,6,7], photothermal therapy [8,9], temperature sensing [10], laser [11,12], bioimaging [13,14] and anti-counterfeiting [2,15,16]. Ln^3+^-doped materials with tunable emission colors as security inks have been one of most commonly utilized methods for high-level anti-fake due to its difficult duplication and tunable luminescence properties [15,17,18]. The emission colors from near-ultraviolet (UV) to NIR region have been realized in Ln^3+^-doped particles by adjusting the types and concentrations of emitters and sensitizers [19,20,21]. For example, the Eu^3+^ ions could emit visible color under UV illumination, which is applied to the anti-counterfeiting purpose in banks [22]. This approach of photoluminescence process is downshifting (DS). The well-known upconversion (UC) rare earth pairs, which can produce intense green, red, violet emissions using 980 nm excitation, are Yb^3+^/Tm^3+^ (Er^3+^, Ho^3+^) systems [5,13,15,22,23]. The dual-mode emission with different emission colors in a single microcrystal could provide an opportunity for higher-level anti-counterfeiting. Moreover, in order to decrease the affection of unnecessary luminescence quenching, these UC and DS Ln^3+^ ions dopants are usually doped into the core and shell layer, respectively. This design disturbs the interaction between these lanthanide ions, and is beneficial to overcome the luminescence quenching [1,13,22].

Among the different inorganic host matrices like oxides, fluorides, and chlorides, the inorganic fluorides are chosen as efficient host materials for both UC and DS luminescence to achieve multicolor tuning. The selection is attributed to their low phonon energy and high stability [3,22,23,24]. Hexagonal-phase NaYF_4_ is not only considered as excellent UC host materials, but also offers efficient DS emission of Ln^3+^ ions such as Tb^3+^ and Eu^3+^, hence generating bright green and red DS emission in the visible region [25,26]. Thus, recently, the Tb^3+^ and Eu^3+^ co-doping in host has aroused many researchers’ interest due to tunable DS emission colors [19,27,28,29]. However, in these literatures, quite little attention has been paid to Tb-Eu co-doped core-shell-structured nanoparticles for dual-mode emission. Furthermore, only core-shell nanoparticles have been investigated to realize tunable emission colors, while studies on hetro- and homogenous microrods structures are still indispensable [3,30,31].

Herein, we propose a strategy to synthesize uniform β-NaYF_4_ microrods that are composed of NaYF_4_:Yb/Er(Tm) core and NaYF_4_:Ce/Tb/Eu shell for achieving dual-mode anti-counterfeiting. These dual-mode core-shell microrods can generate intense multicolor (green, yellow, orange and red) depending on the pump wavelengths (NIR or UV light), the concentration of dopants in shells and detection delay time. Furthermore, due to their microscale dimensions, the materials not only retain higher luminescence efficiency but also have a less complicated process, and lower costs due to lack of filter and outmost layers. Therefore, these materials present adjustable color that could be used as multicolor labels to improve the multimode anti-fake level.

## 2. Materials and Methods

### 2.1. Materials

Y(NO_3_)_3_·6H_2_O (99.99%), Yb(NO_3_)_3_·5H_2_O (99.99%), Er(NO_3_)_3_·6H_2_O (99.99%), Tb(NO_3_)_3_·6H_2_O (99.99%), Eu(NO_3_)_3_·6H_2_O (99.99%), Ce(NO_3_)_3_·6H_2_O (99.99%), NaOH (>98%) were supplied by the HWRK Chemical Co. Ltd., Beijing, China. NH_4_F was supplied by Damao Chemical Reagent Factory, Tianjin, China. NaF was supplied by Jiangtian Chemical Technology Co. Ltd., Tianjin, China. Ethylenediaminetetraacetic acid disodium salt (EDTA-2Na) were supplied by Aladdin Chemical Reagent Co. Ltd., Shanghai, China. Oleic acid (OA), and ethanol were supplied by SanJiang Chemical Technology Co. Ltd., Tianjin, China. All of the chemicals used in this study were of analytical grade and used in the original condition without any further purification.

### 2.2. Preparation of β-NaYF_4_:Yb/Er Microrods

The β-NaYF_4_:Yb/Er microrods were synthesized following a previous approach in the literature [17].

### 2.3. Preparation of Seeding Microrods

All the as-prepared β-NaYF_4_:Yb/Er microrods could be used as seeds after surface treatment. The seeds were prepared following a previous approach in the literature [18,32].

### 2.4. Sequential of Core-Shell-Structured Microrods

The core-shell-structured microrods were synthesized with EDTA-2Na as a chelating agent. The EDTA-2Na (4.70 mL) solution was mixed with the rare earth aqueous solution Ln(NO_3_)_3_ (1.875 mL, 0.2 M; Ln = Ce^3+^, Tb^3+^, and Eu^3+^). After vigorously stirring, the NH_4_F (5 mL, 2 M), and NaF (10 mL, 0.5 M) were added. Then, the HCl (1.875 mL, 2 M), HNO_3_ (1.875 mL, 15 wt%) and the seed crystals (0.094g) were mixed. The resulting mixture was stirred for 40 min and then transferred into a 50 mL Teflon-lined autoclave for heating at 220 °C for 700 min. The samples were spectively centrifuged and washed with deionized (DI) water and ethanol 3 times.

### 2.5. Characterization

X-ray diffraction (XRD) patterns in the 2*θ* ranging from 10 to 80 were measured by the D/max-2500 X-ray diffractometer equipped with graphite-monochromatized Cu Kα radiation (λ = 1.54056 Å) (Riagaku Co. Ltd., Tokyo, Japan). The morphologies of the products were recorded by an scanning electron microscopy (SEM) on ZEISS MERLIN Compact (Carl Zeiss, Oberkochen, Germany) operating at 3 kV. Energy dispersive X-ray spectrometer (EDS) and scanning transmission electron microscopy (STEM) images were performed on the Tecnai G2 F30 transmission electron microscope (FEI, Hillsborough, OR, USA). The UC and DS emission spectra of samples were recorded on a Horiba Fluorolog-3 luminescence spectrometer (Horiba, Edison, NJ, USA) using a 980 nm continuous wave laser. The DS luminescence delay time and time-resolved emission spectra (TRES) were recorded on an Edinburgh FSP-920 fluorescence spectrometer (Edinburgh Instruments, Livingston, Scotland, UK) with the excitation of pulsed Xeon lamp at 252 nm and 395 nm. All the measurements were performed at room temperature.

## 3. Results and Discussion

**Figure 1:**
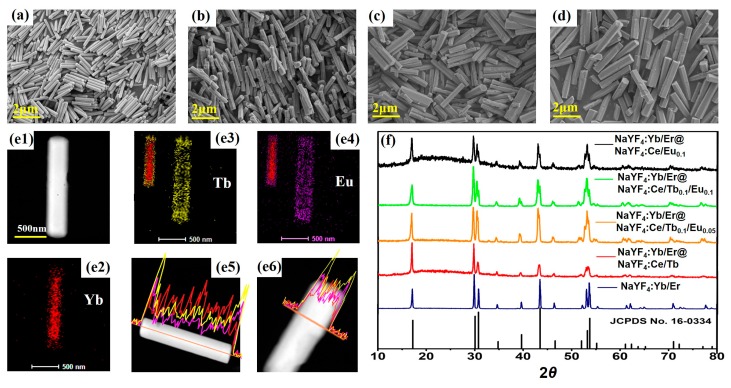
(**a**–**d**) SEM imaging of the samples, NaYF_4_:Yb/Er, NaYF_4_:Yb/Er@NaYF_4_:Ce/Tb_0.1_, NaYF_4_:Yb/Er@NaYF_4_:Ce/Tb_0.1_/Eu_0.05_, NaYF_4_:Yb/Er@NaYF_4_:Ce /Eu_0.1_ microrods. (**e1**) The STEM image of the NaYF_4_:Yb/Er@NaYF_4_:Ce/Tb_0.1_/Eu_0.05_ microrods. (**e2**–**e4**) are the element mappings of Yb, Tb and Eu in a single NaYF_4_:Yb/Er@NaYF_4_:Ce/Tb_0.1_/Eu_0.05_ microrod, respectively. The insets in Figure (**e3**,**e4**) are the element composition images of Yb-Tb and Yb-Eu, respectively. (**e5**,**e6**) are line scans of the element distributions of Yb, Tb and Eu in the single microrod along the axial direction and radial direction, respectively. The red, yellow, and purple lines represent the Yb, Tb and Eu elements, respectively. (**f**) XRD patterns of the samples.

The lanthanide-doped β-NaYF_4_ core-shell microcrystals that are composed of NaYF_4_:Yb/Er as the core and NaYF_4_:Ce/Tb/Eu as the shell for dual-mode emission (UC and DS) were fabricated by hydrothermal reaction. The NaYF_4_:Yb/Er core microrods served as seed microrods after acid cleaning for the shell growth, resulting in the formation of core-shell NaYF_4_:Yb/Er@NaYF_4_:Ce/Tb/Eu microcrystals. The morphologies of materials are displayed by SEM images (Figure 1a–d). Figure 1a shows that the image of seed crystals, with a length and a diameter of about 1.20 and 0.21 μm, respectively. Figure 1b–d indicate the SEM images of NaYF_4_:Yb/Er@NaYF_4_:Ce/Tb_0.1_, NaYF_4_:Yb/Er@NaYF_4_:Ce/Tb_0.1_/Eu_0.05_, and NaYF_4_:Yb/Er@NaYF_4_:Ce /Eu_0.1_ microrods, respectively. The size distribution analysis of all samples are displayed in Figure S1 (Supplementary Materials), indicating the NaYF_4_:Ce/Tb/Eu active shells are grown onto the surface of NaYF_4_:Yb/Er core microrods. Furthermore, the mappings and line scans of elemental distribution are shown in Figure 1e2–e6. Obviously, the element signal intensity varies with the position of the core-shell microrods. Figure 1e2,e5,e6 reveal Yb is uniformly distributed in the core, and Figure 1e3–e6 describe Tb and Eu are uniformly distributed in the shell. This is consistent with the designed element distribution, and further validates the synthesis of core-shell structure. The crystalline seed rods and core-shell microrods can be confirmed by the XRD patterns. As shown in Figure 1f, all the peaks of samples can be indexed to the standard pattern of hexagonal-phase NaYF_4_ microrods (JSPDS No. 16-0334), indicating the formation of pure hexagonal-phase NaYF_4_ microrods for the core and core-shell-structured samples.

**Figure 2:**
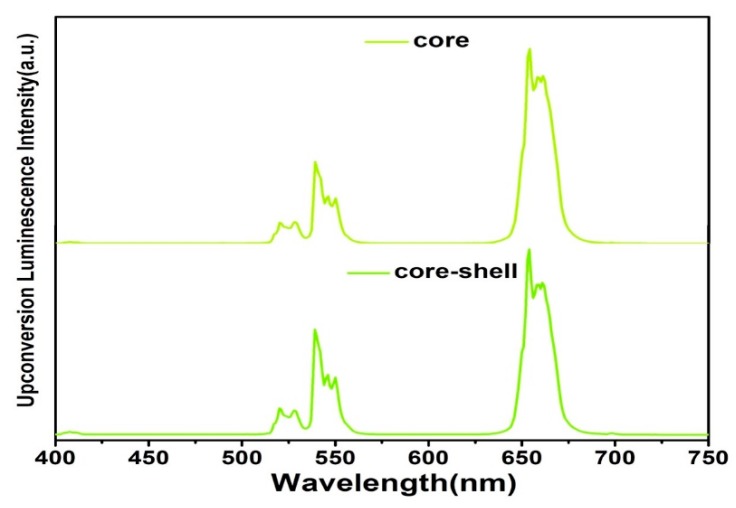
Emission spectra of the seed microcrystals (NaYF_4_:Yb/Er, upper) and core-shell microrods (NaYF_4_:Yb/Er@NaYF_4_:Ce/Tb_0.1_/Eu_0.1_, lower) under 980 nm excitation.

The UC luminescence spectra of samples (NaYF_4_:Yb/Er and NaYF_4_:Yb/Er@NaYF_4_:Ce/Tb_0.1_/Eu_0.01_) are illustrated in Figure 2. Under excitation at 980 nm, the core and core-shell microrods all exhibit three emission bands, which stem from the ^2^H_11/2_→^4^I_15/2_ (523 nm), ^4^S_3/2_→^4^I_15/2_ (541 nm) and ^4^F_9/2_→^4^I_15/2_ (656 nm) transitions from Er^3+^. This result indicates coating the shell hardly hinder the UC process of the seed crystals. However, it is easily noticed that the green-to-red intensity ratio of core-shell microrods is higher than that of core microrods. The Commission Internationale de l’Eclairage (CIE) coordinates of core and core-shell microrods are (0.422, 0.563) and (0.367, 0.611), respectively (Figure S2). Our group has reported that the UC luminescence intensity of the core-shell microrods decreases after shell coating modification [15,32].

**Figure 3:**
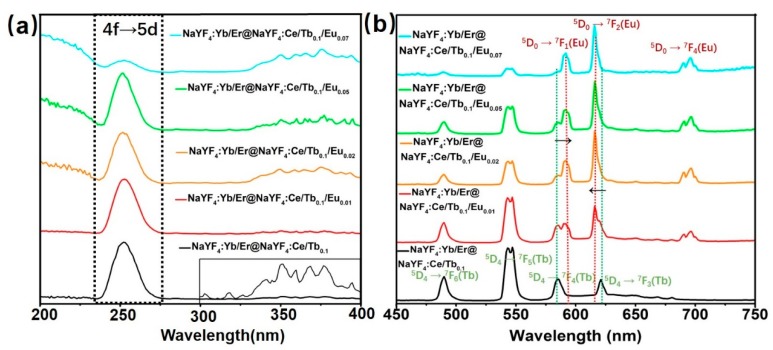
(**a**) Photoluminescence excitation (PLE) spectra of core-shell-structured microrods monitored at 544 nm from Tb^3+^. The inset is the enlarged excitation spectrum of the sample from 300 nm to 400 nm. (**b**) Emission spectra of corresponding samples at 252 nm excitation under the same condition.

**Figure 4:**
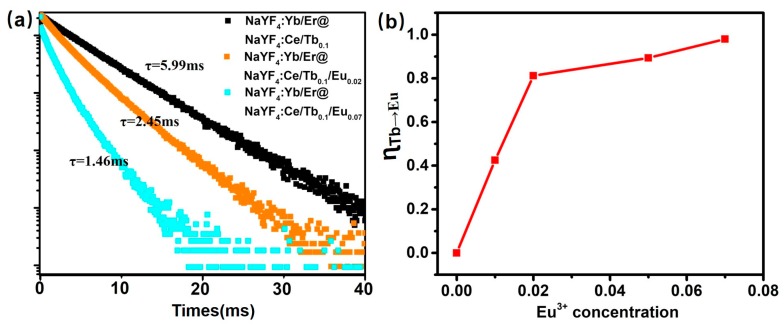
(**a**) Downshifting luminescence delay curve of core-shell-structured microrods at 544 nm with various doping concentrations of Tb^3+^ and Eu^3+^. (**b**) Energy transfer efficiency from Tb^3+^ to Eu^3+^ in NaYF4:Yb/Er@NaYF_4_:Ce/Tb_0.1_/Eu microrods doped with various Eu concentrations.

**Figure 5:**
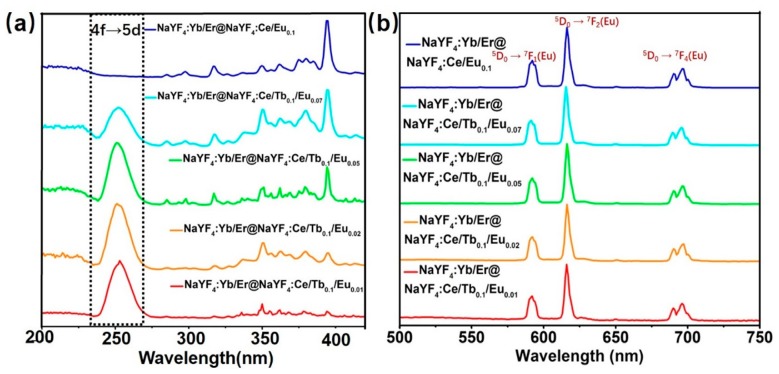
(**a**) Photoluminescence excitation spectra of core-shell-structured microrods monitored at 615 nm from Eu^3+^. (**b**) Emission spectra of corresponding samples at 395 nm excitation under the same condition.

The DS luminescence spectra of the core-shell microrods under UV light are shown in Figure 3, Figure 4 and Figure 5. Figure 3a shows the photoluminescence excitation spectra of core-shell microrods monitored at 544 nm from Tb^3+^. The shell doped with Ce^3+^ and Tb^3+^ ions shows one main absorption band peak at 252 nm, which results from 4f→5d transition from Ce^3+^ ions [21,22,26]. The corresponding emission spectra of NaYF_4_:Yb/Er@NaYF_4_:Ce/Tb/Eu microrods are displayed in Figure 3b when excited at 252 nm directly. The emission spectra are recorded by characteristic transition for both Tb^3+^ and Eu^3+^ in the visible region [25,26,27,28]. The emission peaks at 489, 544, 584 and 620 nm due to electronic transition from Tb^3+^ ions (^5^D_4_→^7^F_J_ (J = 6, 5, 4, and 3)) are observed. This indicates that the Ce^3+^ ions absorb UV light and transfer energy to neighboring Tb^3+^ ions, resulting in the luminescence emission from Tb^3+^ ions. As discussed by previous reports [19,27,28,29], the Tb^3+^ and Eu^3+^ ions are utilized together to achieve the tunable color and obtain bright multicolor emission. Therefore, we reported the NaYF_4_:Ce/Tb/Eu shells are grown on the UC microrods. With the increasing concentration of Eu^3+^ ions in shells, the intensity of emission bands from Tb^3+^ ions suffer from quenching, whereas the intensity of the Eu^3+^ is enhanced (Figure 3b). Figure 3b shows that the emission spectra exhibit the strong emission bands of Eu^3+^ ions at 590, 615 and 695 nm when the concentration of Eu^3+^ is above 2 mol%. The strongest emission peak is 615 nm. The three emission bands of Eu^3+^ are attributed to ^5^D_0_→^7^F_J_ (J = 1, 2, and 4) transition, respectively. This result indicates that the Tb^3+^ ions act as intermediate ions to achieve Ce^3+^→Tb^3+^→Eu^3+^ energy transfer.

To comprehend the energy transfer, the decay curves of Tb^3+^ emission at 544 nm and energy transfer efficiency (*ɳ_Tb-Eu_*) from Tb^3+^ to Eu^3+^ of core-shell microrods are presented in Figure 4. Figure 4a demonstrates the lifetime curve of the NaYF_4_:Yb/Er@ NaYF_4_:Ce/Tb_0.1_/Eu_x_ (x = 0, 0.02, and 0.07) under a pulsed excitation wavelength of 252 nm. The effective lifetime of the excited state was calculated according to the equation: τ_eff_ = ʃI*(t)*d*t*/I_0_, where I(t) is the luminescence intensity at time t and I_0_ represents the maximum intensity [33]. It is worthy to notice here that by increasing the Eu^3+^ concentration from 0 to 7 mol% in the shell, the lifetime of Tb^3+^ emission at 544 nm decreases from 5.99 ms to 1.46 ms. For more thoroughly investigating the energy transfer of Tb^3+^ and Eu^3+^ ions, the *ɳ_Tb-Eu_* was also calculated by the following equation [21,29]:

(1) $$\eta_{Tb\to Eu}=1-\frac{I}{I_{0}}$$

where *I* and *I*_0_ are the emission intensities of the Tb^3+^ ions at 544 nm with and without Eu^3+^ ions, respectively. The *ɳ_Tb-Eu_* was calculated as a function of doping concentration of Eu^3+^, as shown in Figure 4b. It is found that the *ɳ_Tb-Eu_* increases rapidly with the increasing Eu^3+^ concentration. The *ɳ_Tb-Eu_* increases to 97.9% at NaYF_4_:Yb/Er@NaYF_4_:Ce/Tb_0.1_/Eu_0.7_ microrods. The phenomenon indicates efficient Tb^3+^→Eu^3+^ energy transition and achievement of the tunable color emission. The CIE coordinates of NaYF_4_:Yb/Er@NaYF_4_:Ce/Tb_0.1_/Eu microrods shift from (0.414, 0.517) to (0.488, 0.383) by tuning the concentration of Eu^3+^ from 0 to 0.07 mol% (Figure S3). This means the emission color of samples shifts from yellow-green to orange under UV lamp (λ = 252 nm) excitation.

Besides, DS emission from Eu^3+^ ions in core-shell microrods is also obtained under 395 nm excitation directly. The excitation spectra and emission spectra of core-shell microrods by doping Eu^3+^ in the shells are shown in Figure 5. The PLE spectra of samples, monitoring emission at 615 nm (Figure 5a), contain two main peaks at 252 nm and 395 nm. It is noticed that the PLE spectra of samples by co-doping Tb^3+^ and Eu^3+^ ions include all spectral features for co-doped Ce/Tb and Ce/Eu ions (Figure 3a and Figure 5a). The excitation band centered at 252 nm suggests the energy transition process from Ce^3+^ to Eu^3+^ via Tb^3+^, as discussed above. As the doping concentration of Eu^3+^ increases in the shells, the emission band peak at 395 nm is dominant, which is attributed to ^7^F_0_→^5^L_6_ transition of Eu^3+^ ions (Figure 5a). According to Figure 5a, NaYF_4_:Yb/Er@NaYF_4_:Ce/Tb/Eu microrods can be excited directly by 395 nm light, which is the characteristic excitation band of Eu^3+^, as previously reported [16,34]. The corresponding emission spectra of the samples at 395 nm excitation are shown in Figure 5b. Independent of the doping of Ce^3+^ and Tb^3+^ ions, the emission spectra profiles are the same (Figure 5b, and Figure S4), consisting of characteristic peaks of Eu^3+^ due to ^5^D_0_→^7^F_J_ (J = 1, 2, and 4) transition. The luminescence intensity increases with the Eu content (Figure S4). However, the intensity ratios of the emission bands change as a function of Eu^3+^ concentration in samples under the same excitation condition (Figure S5). This is ascribed to the influence of symmetry of host lattice on ^5^D_0_→^7^F_2_ transition [19]. Hence, the emission color of core-shell microrods gradually changes from orange to red, and the corresponding CIE color coordinates of these samples are shown in Figure S3b. The coordinates shift from (0.525, 0.457) to (0.459, 0.437). As a result, the NaYF_4_:Yb/Er@NaYF_4_:Ce/Tb/Eu microrods can emit visible light from yellow-green to red color by adjusting the concentrations of Tb^3+^ and Eu^3+^ in shells and UV excitation wavelengths.

Owing to the coexistent DS emission from Tb^3+^ and Eu^3+^ under 252 nm excitation, the separation of DS emission between Tb^3+^ and Eu^3+^ could be achieved by time-resolved luminescence detection technique, which is ascribed to their different decay lifetimes (Figure S6). In Figure S6a, it is noticed that the decay time of Eu^3+^ emission is longer than that of Eu^3+^ emission in previous reported. This is because the lifetime of the excited state is affected by the host and the synthesis procedure. The longer lifetime of Eu^3+^ ions is obtained when the Eu^3+^ ions are in a more symmetric environment [19]. On the other hand, the transient curve of Eu^3+^ emission exhibits rise and decay components, which indicates the energy transfer process [35]. The curve can be well fitted with the following equation [36]:

(2) $$I(t)=-Ae^{-t/\tau_{r}}+B_{1}e^{-t/\tau_{1}}+B_{2}e^{-t/\tau_{2}}$$

where *A*, *B*_1_ and *B*_2_ are emission intensity constants, and *τ_r_* and *τ_1,2_* represent the rise and delay time, respectively. The calculated rise time of the NaYF_4_:Yb/Er@NaYF_4_:Ce/Tb_0.1_/Eu_0.05_ microrods is 1.97 ms. Furthermore, we calculated rise time of other samples with various concentrations of Eu^3+^ ions. The rise time of the emission at 615 nm decreases with the increasing concentration, as shown in Figure S6b, which is attributed to the distance of energy transition becoming shorter with increasing concentration of Eu^3+^ ions [34,37,38].

**Figure 6:**
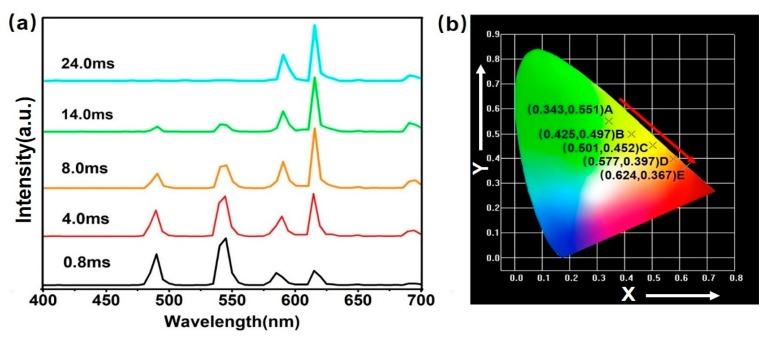
(**a**) Time-resolved emission spectra for NaYF_4_:Yb/Er@NaYF_4_:Ce/Tb_0.1_/Eu_0.05_ microrods at 252 nm excitation. (**b**) The CIE coordinates of the sample at various delay times.

Figure 6a shows the time-resolved emission spectra (TRES) for NaYF_4_:Yb/Er@NaYF_4_:Ce/Tb_0.1_/Eu_0.05_ microrods by setting various delay times at 252 nm excitation. The long delay time emission of Eu^3+^ could be distinguished from that of Tb^3+^, which means in the long delay time (>8 ms), the whole DS emission is dominated by the red emission from Eu^3+^ using UV lamp (252 nm) excitation. At the same time, the emission from Tb^3+^ is prominent by setting the short delay time (0.8–8 ms). This indicates that the emission multicolor outputs, changing from green to red region, in the time domain from NaYF_4_:Yb/Er@NaYF_4_:Ce/Tb_0.1_/Eu_0.05_ microrods could be achieved by setting suitable delay time. The corresponding CIE coordinates are shown in Figure 6b. The DS multicolor outputs (time-resolved and pump-wavelength-induced DS) coupled with the UC mode could pave the way for the anti-counterfeiting fields.

**Figure 7:**
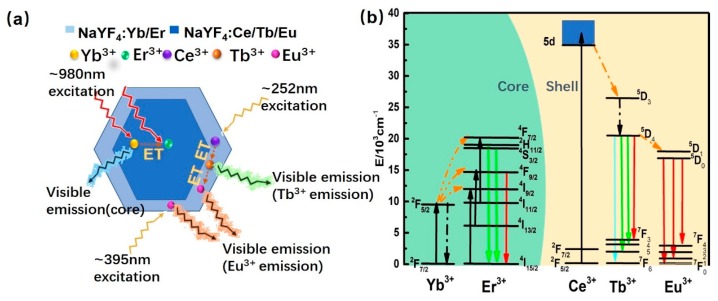
(**a**) Schematic illustration of NaYF_4_:Yb/Er@NaYF_4_:Ce/Tb/Eu core-shell microcrystals to achieve the dual-mode emission. (**b**) Proposed schematic diagram of the energy transfer processes for dual-mode emission in the core-shell microrods.

We successfully fabricated the core-shell microrods that can realize dual-mode (UC and DS) luminescence from Er^3+^, Tb^3+^ and Eu^3+^ ions. The schematic of the prepared core-shell microrods is shown in Figure 7a. Efficient UC luminescence can be obtained with 980 nm laser irradiation, giving rise to the emission of green, red or yellow colors. As the shell is doped with Ce^3+^/Tb^3+^/Eu^3+^ ions, Ce^3+^ ion will act as a sensitizer and promote the energy transition to activators (Tb^3+^ ions). Podhorodecki [25] and Jang [26] have confirmed the excitation of Eu ions through Tb ions due to the similar excitation spectra recorded for emission of Eu^3+^ ions between NaYF_4_:Tb,Eu and NaYF_4_:Eu samples. It indicates that the excitation energy can be transferred from Ce^3+^ to Eu^3+^ ions. Meanwhile, red DS emission from Eu^3+^ ions is also obtained under 395 nm excitation.

The luminescence mechanism for NaYF_4_:Yb/Er@NaYF_4_:Ce/Tb/Eu core-shell microcrystals system is displayed in Figure 7b. The UC luminescence can be obtained from core microrods at 980 nm excitation, which stems from the 4f electronic transition of Er^3+^ [22,32]. Under 980 nm excitation, Yb^3+^ ions transfer energy to Er^3+^ after absorbing excitation energy. The excitation levels (^2^H_11/2_/^4^S_3/2_, ^4^F_9/2_ states) of Er^3+^ are populated via two successive energy transition processes (Figure S7), and green (~540 nm) and red (~654 nm) emissions are generated. For the Ce^3+^-Tb^3+^-Eu^3+^ system in shells, the DS emission can be achieved. The Ce^3+^ ions absorb the external UV light (~252 nm) due to 4f→5d transition and then transfer to ^5^D_4_ state of Tb^3+^ ions efficiently (Figure 4). Afterwards, the green emission is obtained through the radiative transition from ^5^D_4_ state to ^7^F_J_ (J = 3, 4, 5, and 6) states. With Eu^3+^ ions doping, Tb^3+^→Eu^3+^ energy transfer occurs, and a part of excited energy from ^5^D_4_ state of Tb^3+^ ions shifts to ^5^D_0_ state. Finally, the red emission is generated ascribed to ^5^D_0_→^7^F_J_ (J = 1, 2, 4) transition. In addition, the red emission from Eu^3+^ can also be obtained using 395 nm excitation straightforwardly.

## 4. Conclusions

We have successfully designed an approach to achieve UC and DS luminescence simultaneously in core-shell microrods (NaYF_4_:Yb/Er@NaYF_4_:Ce/Tb/Eu). This approach helps confine the activators to different regions, hence overcoming the effect of the cross-relaxations between activators, and meanwhile minimizing the effect of surface quenching without an outmost layer. The NaYF_4_:Yb/Er@NaYF_4_:Ce/Tb/Eu microrods could achieve multicolor emission depending on excitation wavelengths (980 nm, 252 nm, and 395 nm), the concentration of Eu^3+^ in shells and detection delay time. Under the 980 nm excitation, the core of Yb/Er system for the UC process emits yellow color. While, the shell of Ce/Tb/Eu systems for the DS process emits visible light from yellow-green to red color by adjusting the concentrations of Eu^3+^ and UV excitation wavelengths (252 nm, and 395 nm). Moreover, because of the different decay lifetime of Tb^3+^ and Eu^3+^ ions at their characteristic emission wavelengths, the emission multicolor outputs, changing from green to red region, in the time domain from NaYF_4_:Yb/Er@NaYF_4_:Ce/Tb_0.1_/Eu_0.05_ microrods could be realized by optimizing delay time. Due to high UC/DS efficiency and dual-mode luminescence with multicolor emission, the NaYF_4_:Yb/Er@NaYF_4_:Ce/Tb/Eu microrods have promising applications in displays and anti-counterfeiting fields.

## Supplementary Materials

The following are available online at http://www.mdpi.com/2079-4991/8/12/1023/s1, Figure S1: Size distribution analysis of the NaYF_4_: Yb/Er @NaYF_4_:Ce/Tb/Eu microcrystals along the axial direction (**a**) and radial direction (**b**) collected at various doping concentration ratios of Tb/Eu, Figure S2: The CIE chromaticity coordinates of the emission of the seed microcrystals (NaYF_4_:Yb/Er) and core-shell microrods (NaYF_4_:Yb/Er@NaYF_4_:Ce/Tb_0.1_/Eu_0.1_) under 980 nm excitation, Figure S3: CIE chromaticity coordinates of the emission of samples with various doping concentrations of Tb^3+^ and Eu^3+^. The direction of the arrow denotes the changing of emission colors, Figure S4: Pump-power-dependent downshifting luminescence spectra of the core-shell-structured microrods with various doping concentration ratios of Tb/Eu under 395 nm excitation, Figure S5: Relative intensity of different emission bands in core-shell microrods under 395 nm irradiation, Figure S6: (**a**) Delay curves of NaYF_4_:Yb/Er@NaYF_4_:Ce/Tb_0.1_/Eu_0.05_ microrods at 544 nm and 615 nm under 252 nm excitation, (**b**) rise time of emission at 615 nm for NaYF4:Yb/Er@NaYF_4_:Ce/Tb_0.1_/Eu microrods doped with different Eu concentrations, Figure S7: (**a**) Pump-power-dependent upconversion luminescence spectra of the core-shell-structured microrods (NaYF_4_:Yb/Er@NaYF_4_:Ce/Tb_0.1_/Eu_0.01_) using 980 nm excitation and (**b**) corresponding log-log plots of upconversion emission intensity versus excitation power.
