# Supplementary material for: Simultaneous Dual-Mode Emission and Tunable Multicolor in the Time Domain from Lanthanide-Doped Core-Shell Microcrystals
Source: Nanomaterials (Basel). 2018 Dec 7;8(12):1023. doi: 10.3390/nano8121023 (PMC6316006; doi:10.3390/nano8121023)
Supplement: Supplementary file 1 [file nanomaterials-08-01023-s001.pdf]

Supporting Information

Simultaneous Dual-mode Emission and Tunable Multicolor in the Time Domain from Lanthanide-doped Core-shell Microcrystals

Dandan Ju [1,2], Feng Song [1,2,*], Adnan Khan [1,2], Feifei Song [1], Aihua Zhou [1,2], Xiaoli Gao [1,2], Huimin Hu [1,2], Xu Sang [1,2] and Victor Zadkov [3]

[1] School of Physics & The Key Laboratory of Weak Light Nonlinear Photonics, Ministry of Education, Nankai University, Tianjin, 300071, China; judandan@mail.nankai.edu.cn (D.J.); adnanphyzx@mail.nankai.edu.cn (A.K.); songff@mail.nankai.edu.cn (F.S.); zhouihua@mail.nankai.edu.cn (A.Z.); 1120180066@mail.nankai.edu.cn (X.G.); 2120170177@mail.nankai.edu.cn (H.H.); 2120180202@mail.nankai.edu.cn (X.S.); zadkov@isan.troitsk.ru (V.Z.)

[2] Collaborative Innovation Center of Extreme Optics, Shanxi University, Taiyuan, Shanxi, 030006, China

[3] The Institute of Spectroscopy of the Russian Academy of Sciences, Moscow, 142190, Russia

* Correspondence: fsong@nankai.edu.cn; Tel.: +86-022-23502572

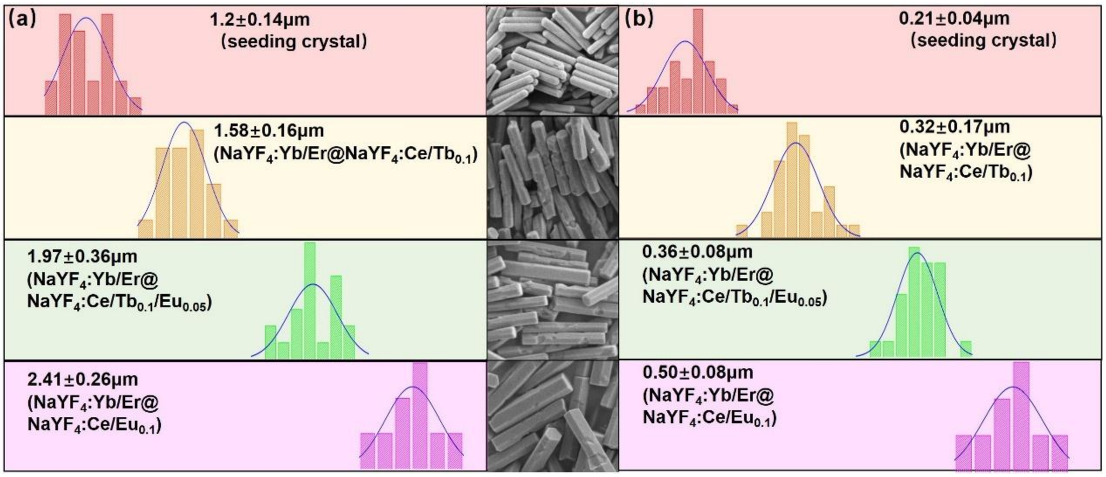

Figure S1. Size distribution analysis of the $NaYF_4$: Yb/Er@$NaYF_4$:Ce/Tb/Eu microcrystals along the axial direction (**a**) and radial direction (**b**) collected at various doping concentration ratios of Tb/Eu.

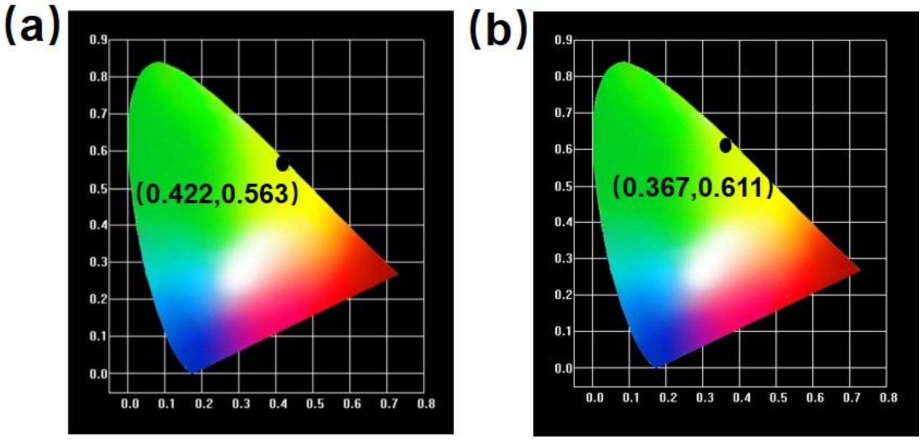

Figure S2. The CIE chromaticity coordinates of the emission of the seed microcrystals ($NaYF_4$:Yb/Er) and core-shell microrods ($NaYF_4$:Yb/Er@$NaYF_4$:Ce/$Tb_{0.1}$/$Eu_{0.1}$) under 980 nm excitation, respectively.

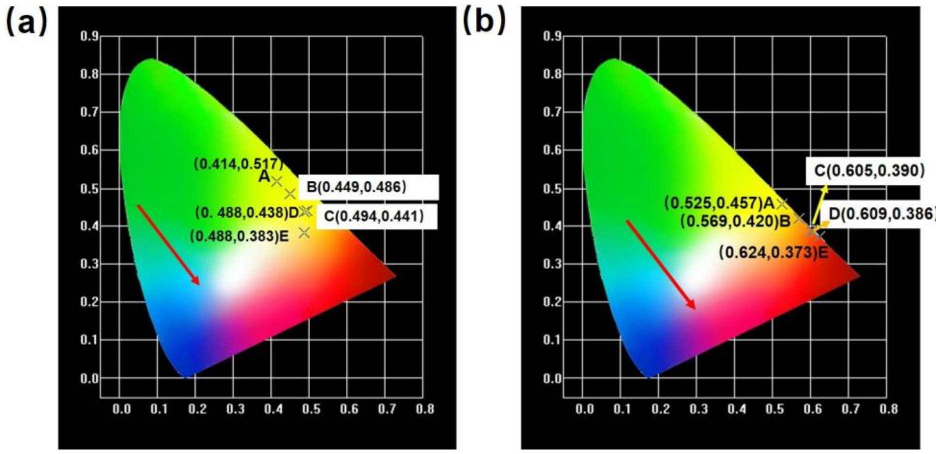

Figure S3. CIE chromaticity coordinates of the emission of samples with various doping concentrations of Tb^{3+} and Eu^{3+}. The direction of the arrow denotes the changing of emission colors.

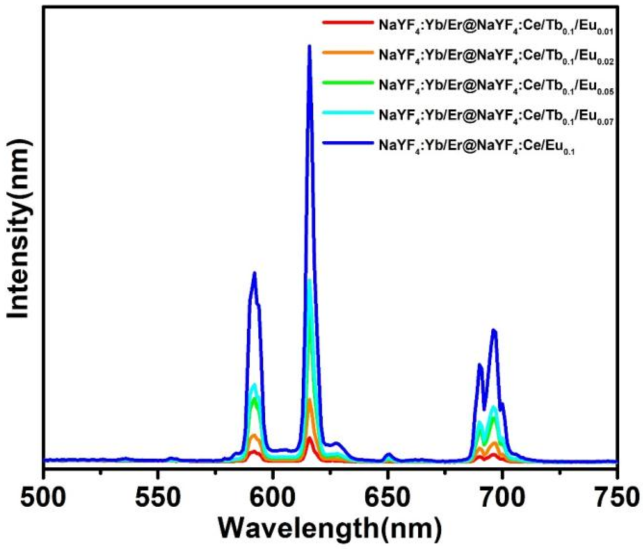

Figure S4. Pump-power-dependent downshifting luminescence spectra of the core-shell structured microrods with various doping concentration ratios of Tb/Eu under 395 nm excitation.

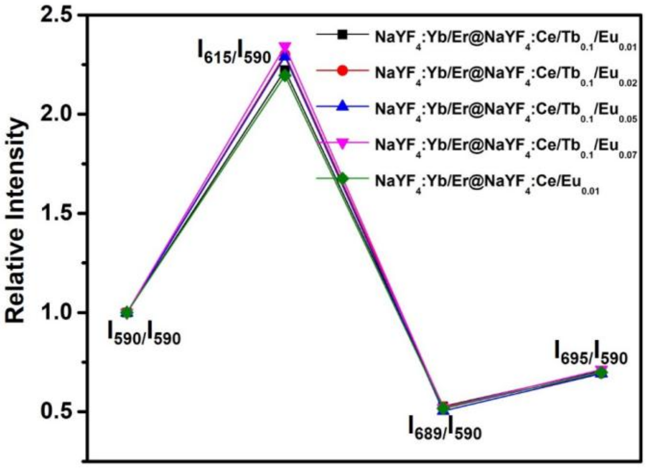

Figure S5. Relative intensity of different emission bands in core-shell microrods under 395 nm irradiation.

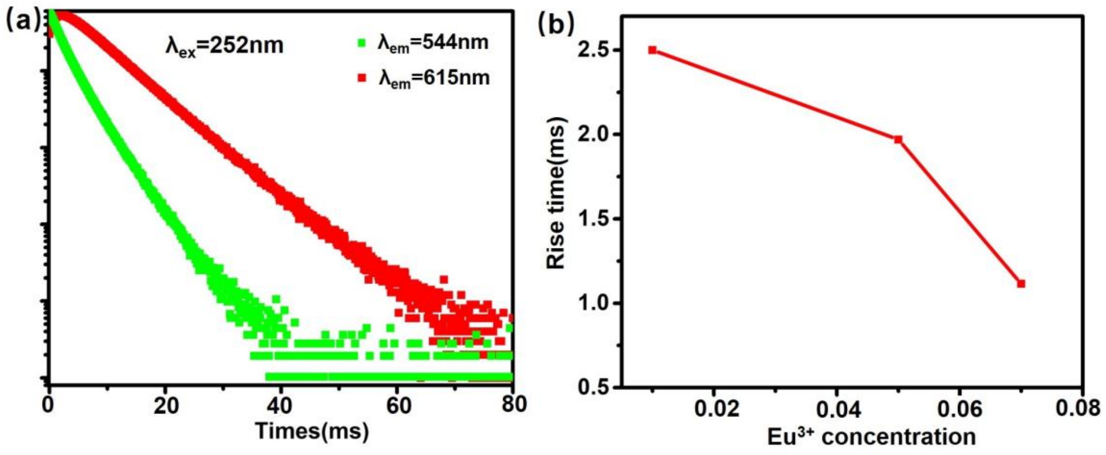

Figure S6. (**a**) Delay curves of NaYF4:Yb/Er@NaYF4:Ce/Tb0.1/Eu0.05 microrods at 544 nm and 615 nm under 252 nm excitation, (**b**) rise time of emission at 615 nm for NaYF4:Yb/Er@ NaYF4:Ce/Tb0.1/Eu microrods doped with different Eu concentrations.

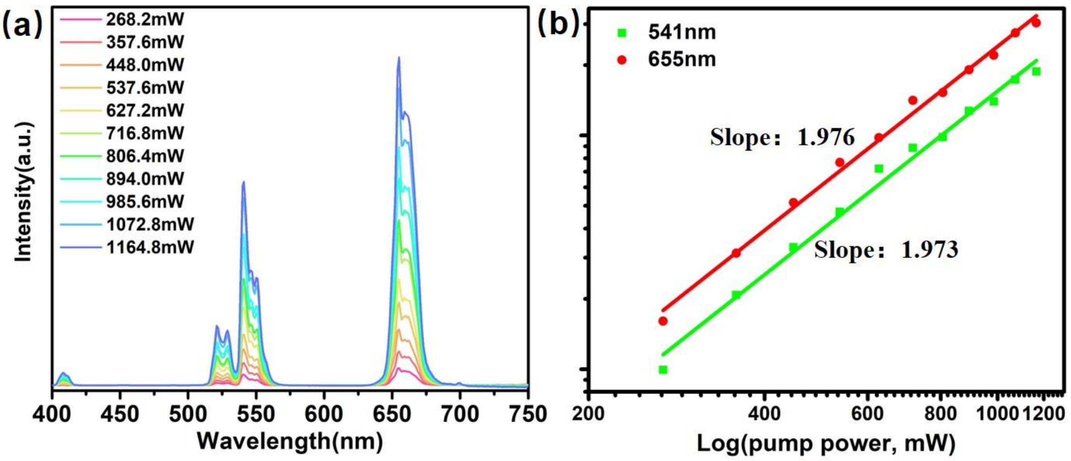

Figure S7. (**a**) Pump-power-dependent upconversion luminescence spectra of the core-shell-structured microrods (NaYF4:Yb/Er@NaYF4:Ce/Tb0.1/Eu0.01) using 980 nm excitation and (**b**) corresponding log-log plots of upconversion emission intensity versus excitation power.

References

1. Back, M.; Marin, R.; Franceschin, M.; Sfar Hancha, N.; Enrichi, F.; Trave, E.; Polizzi, S. Energy transfer in color-tunable water-dispersible Tb–Eu codoped CaF_2 nanocrystals. *J. Mater. Chem. C* **2016**, *4*, 1906–1913. DOI: 10.1039/c5tc03355a.
2. Liu, Y.; Tu, D.; Zhu, H.; Li, R.; Luo, W.; Chen, X. A strategy to achieve efficient dual-mode luminescence of Eu^{3+} in lanthanides doped multifunctional $NaGdF_4$ nanocrystals. *Adv. Mater.* **2010**, *22*, 3266–3271. DOI: 10.1002/adma.201000128.
3. Dong, H.; Sun, L.D.; Feng, W.; Gu, Y.; Li, F.; Yan, C.H. Versatile spectral and lifetime multiplexing nanoplatform with excitation orthogonalized upconversion luminescence. *ACS Nano* **2017**, *11*, 3289–3297. DOI: 10.1021/acsnano.7b00559.
